# Research on External Insulation Characteristics of Composite Cross-Arm of 10 kV Distribution Network Based on Multi-Factor Aging

**DOI:** 10.3390/polym14071403

**Published:** 2022-03-30

**Authors:** Zhongyuan Zhang, Junwei Qi, Hechen Liu, Wanxian Wang, Mingjia Zhang, Xuan Wu

**Affiliations:** Hebei Provincial Key Laboratory of Power Transmission Equipment Security, School of Electrical Engineering, North China Electric Power University, Baoding 071003, China; hvzzv01@163.com (Z.Z.); jumwencepu@163.com (J.Q.); 220192213050@ncepu.edu.cn (W.W.); 18367683603@163.com (M.Z.); 220192213010@ncepu.edu.cn (X.W.)

**Keywords:** multi-factor aging system, composite cross-arms of distribution networks, electrical external insulation characteristics, silicone rubber sheath, hydrophobicity, scanning electron microscope, FTIR

## Abstract

With the application of the composite cross-arm in power systems, comprehensive anti-aging performance is a key factor to determine whether it can operate safely. In order to study the influence of the operating environment on the external insulation characteristics of composite cross-arms of distribution networks, various aging conditions such as voltage, rain, temperature, humidity, salt fog and ultraviolet light were simulated in a climate chamber based on the real operation conditions of the 10-kV composite cross-arm. A multi-factor aging test of composite cross-arms with two kinds of cross-section shapes (T-shaped and square) was carried out for 5000 h. The change trends of leakage current and flashover voltage of the composite cross-arms before and after aging were analyzed. Finally, the aging mechanism of the silicone rubber sheaths was analyzed to further explain the reasons for the change of external insulation performance of composite cross-arms. The results show that the leakage current rising rate of T-shaped and square composite cross-arms after aging increases significantly, and the minimum flashover voltage decreases to 58.3 kV and 49.502 kV, respectively. The results of FTIR, SEM and hydrophobic angle tests show that, after aging, the performance of the silicone rubber outer sheath material decreases in varying degrees. In general, UV aging has the greatest influence on the external insulation characteristics of composite cross arms. Generally speaking, after 5000 h of multi factor aging, although the external insulation characteristics of the 10-kV composite cross-arm decreases to a certain extent, there is still enough margin to meet the normal operation.

## 1. Introduction

Distribution-network composite cross-arms have good insulation performance, high strength and lightweight characteristics [1,2,3]. The United States, Japan and other countries have carried out research on composite cross-arms as early as the 1960s and formulated the application standards of composite products in the field of transmission towers. China’s distribution network of composite cross-arms is developing rapidly; so far it has entered the key technology application stage. However, after a long time in operation, the composite cross-arm is eroded and damaged by various factors of the natural environment, especially during high salt sea fog in coastal areas, alternating high and low temperatures, ultraviolet radiation, mechanical fatigue of the conductor due to wind deflection and galloping, etc. Various aging factors affect the operation life of the composite cross-arm, but each factor plays a different role. Generally, damp heat aging will lead to failure, cracking and deformation of the interlayer interface of the composite cross-arm’s polymer materials, which will seriously affect the mechanical properties of the materials; electric aging and salt spray erosion for a long period of time will lead to discharge corrosion, burn marks and dirt molecules with high conductivity on the surface of the cross arm, which will weaken the external insulation performance of the composite cross-arm; mechanical aging will cause delamination, wear and cracking of materials; UV irradiation will reduce the hydrophobicity and fade the color of the silicone rubber sheath on the surface of the composite cross-arm. The above phenomena seriously affect the normal operation of the composite cross arm. Therefore, domestic and foreign research institutions have proposed to carry out the quality supervision and service life evaluation of the composite cross-arm, focusing on its mechanical fatigue performance and external insulation aging performance.

At present, the research on composite cross-arms in China and abroad mainly includes the mechanical properties of the cross-arm structure, the characteristics of internal insulation interface and the pollution flashover performance of composite cross-arms [4,5]. With the application and promotion of composite cross-arms in distribution networks, domestic research institutions and scholars have made preliminary attempts to test the accelerated aging of the composite cross-bar under a simulated natural environment. Scholar Rui Ke [6] carried out a flashover test of composite cross-arms after single-factor hygrothermal aging for 1000 h. The results showed that the external insulation performance of composite cross-arms changed little, and the aging resistance was acceptable. Xiong Wu [7] carried out an artificial pollution power frequency voltage test and power frequency rain resistance voltage test on different types of composite cross-bars, and obtained flashover voltage data of composite cross-bars under different external conditions. Linong Wang [8] built the surface potential distribution model of a composite tower after multifactor aging and proposed that anti-aging treatments should be carried out for the parts with high field strength and potential.

At present, the research on composite insulation cross-bars is mostly carried out by cutting small samples, and the aging factors are relatively single. There is a lack of multi-factor aging test research for the full-sized composite cross-bar. To sum up, in this paper, combined with the real operating environment of the composite cross-arm, a 5000-h aging test was carried out in the laboratory on two types of composite cross-bar under different aging factors such as voltage, rain, temperature, humidity, salt spray and ultraviolet light. The influence of multi-factor aging on the leakage current and contamination flashover voltage of the composite cross-arm was explored. In addition, this paper analyzes the deterioration mechanism of the silicone rubber outer sheath on the surface of the composite cross-arm by employing scanning electron microscopy and Fourier transform infrared spectroscopy, and then explains the reasons for the change of the outer insulation properties.

## 2. Materials and Methods

### 2.1. Construction and Test Plan of the Artificial Multi-Factor Aging Test System

#### 2.1.1. Sample Selection

**Figure 1:**
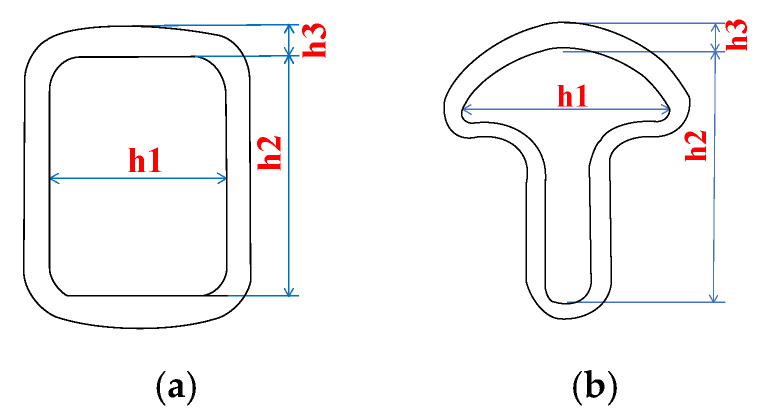
Schematic diagram of the cross-section of the composite cross-arms (**a**) square-shaped; (**b**) T-shaped.

In this test, the widely used square composite cross-arm and T-type composite cross-arm were selected to carry out aging characteristics research, provided by manufacturer A and manufacturer B, respectively. The size of the square composite cross-arm was 32 × 42 × 1750 mm, and the size of the T-shaped composite cross-arm was 35 × 73 × 1860 mm. The schematic diagram of the two composite cross-arms is shown in Figure 1, and the detailed structural parameters are shown in Table 1.

#### 2.1.2. Comprehensive Aging System Construction and Test Plan

**Figure 2:**
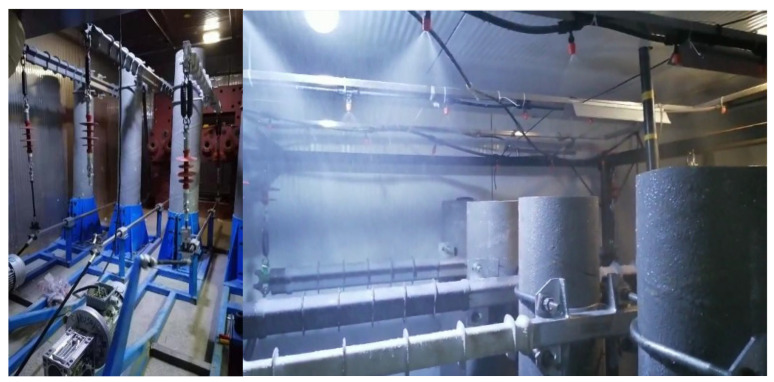
The actual effect diagram of the comprehensive aging system.

This paper refers to the IEC/TR 62730-2012 standard. It combines the actual operating environment of the composite cross-arm to build a set of test systems that can simultaneously carry out voltage, rain, temperature, humidity, mechanical force, salt spray and ultraviolet aging [9]. Table 2 shows the multi-factor aging test scheme. For the test, each cycle is comprised of 24 h, and each cycle is divided into 12 stages, each of which is two hours long. “√” in the table indicates that the corresponding aging factors are applied. Table 3 shows the parameters of each aging subsystem. The temperature, humidity, amount of rain and amount of salt spray simulates the natural climate of subtropical coastal areas. The ultraviolet radiation intensity refers to GBT 16,422.2 Plastic Laboratory Light Source Exposure Test Method (Method A), and its radiation intensity was 550 W/m^2^. The span of the cross-arm was set to 50 m, the wire JKLYJ-10/150 was selected, the cross-sectional area of the wire was 156.41 mm^2^, the horizontal specific load was 209.263 [×10^−3^ N/(m∙mm^2^)] and the vertical-specific load was 40.879 [×10^−3^ N/(m∙mm^2^)]. The horizontal and vertical forces at both ends of the composite cross-arm are calculated according to DL/T 5220-2005 “Technical Specifications for Design of Overhead Distribution Lines of 10 kV and Below”. To simulate the vibration of the composite cross-arm under wind load, this test system uses a crank slider installed on a motor to drive the connecting rod to move horizontally. At the same time, the connecting rod drives the spring, causing the other components to swing back and forth. Periodic mechanical stress was applied to the composite cross-arm at a frequency of 0.6 Hz. Therefore, according to the different cross-section shapes of composite cross-arms and aging factors, the cross-arms of the test samples were divided into six categories. See Table 4 for the specific classification method and cross-arm number. Figure 2 shows the actual effect of multi factor aging of composite cross arm.

#### 2.1.3. Overview of Composite Cross-Arm Samples after Multi-Factor Aging

**Figure 3:**
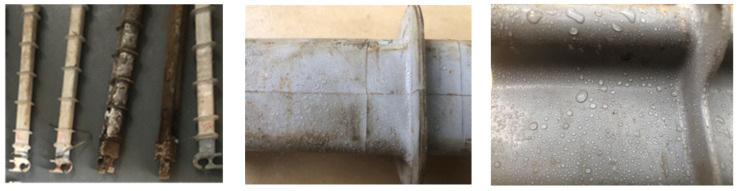
Morphology of two kinds of composite cross-arm samples after multi-factor aging.

The morphology of the two composite cross-arm samples after 5000 h of multi-factor aging is shown in Figure 3. It can be seen from the figure that the macro appearance of the aged composite cross-arm has changed to a certain extent. Part of the umbrella skirt and sheath of the cross-arm turn yellow after ultraviolet irradiation, but the overall shape of the cross-arm has not changed greatly. After further observation, it was found that all cross-arm samples were aged by salt fog and rain; the contaminated particles mixed with salt, soil and dust were attached to the silicone rubber surface; the metal fittings at the end of the composite cross arm were seriously rusted; and a small amount of sheath near the end metal fittings was damaged. It is considered that this is because a small amount of silicone rubber sheath at the end was damaged and cracked after mechanical force fatigue vibration was applied to the composite cross-arm. In order to explore the changes of the external insulation characteristics of the composite cross-arm after multi-factor aging, it is necessary to carry out artificial pollution electrical experiments on it and analyze the microscopic mechanism of the deteriorated composite cross-arm’s silicone rubber sheath to discuss its performance characteristics after aging.

### 2.2. Artificial Pollution Test of the Composite Cross-Arm after Aging

#### 2.2.1. Sample Selection

To analyze the influence of multi-factor aging on the external insulation performance of the composite insulating cross-arm, a pollution flashover test and leakage current test were carried out on the composite insulating cross-arm before and after aging. Contaminants were simulated by soluble NaCl and inert dirt (diatomite). The ash-salt ratio (PNSDD/PESDD) was selected as 5:1 [10]. Considering the climatic conditions in coastal areas, the PESDD/PNSDD was 0.2/1.0 mg/cm^2^. The smearing method adopts the quantitative brushing method. First, the mass of NaCl and diatomite is calculated based on the surface area of the composite cross-arm. After weighing, deionized water was added to form a dirty liquid, and then applied evenly to the surface of the silicone rubber sheath. After smearing the cross-arm, drying takes 20–25 h in the 5 m × 2.5 m × 2 m artificial fog chamber, with the climate chamber temperature set to 25 °C and the humidity set to 55%.

#### 2.2.2. Experiment Method

In this paper, the artificial contamination leakage current of composite cross-arms before and after aging at four voltages (U_0_ = 10/√3, 1.8 U_0_, 2.6 U_0_, 3.5 U_0_) was measured.

The flashover test uses the uniform boost method to determine the flashover voltage value [11,12]. The dried cross-arm sample is wetted with steam mist at a constant rate of 0.05 ± 0.01 kg/(h m^3^) in the artificial fog chamber, and then quickly moved to the indoor high-pressure experimental area after reaching saturation. The voltage is gradually increased at a rate of 2 kV/s until flashover. Each cross-arm is tested five times, and the average value is taken as the 50% flashover voltage (U_50%_) of the cross arm of this aging type. U_50%_ is calculated by the following formula:

(1) $$U_{50\%}=\frac{\overset{N}{\underset{i=1}{\sum }}U_{i}}{N}$$

In the formula, U_50%_ is the composite cross-arm flashover voltage obtained by the uniform boost method, kV; *U_i_* is the *i*th flashover voltage, kV; *N* is the number of tests.

### 2.3. Observation of Discharge Characteristics of the Composite Cross-Arm after Aging

To verify the influence of various section shapes and different aging factors on the discharge characteristics of the composite cross-arm. In this paper, the CoroCAM504 UV imager was used to observe the number of light spots as the voltage of the composite cross-arm is increased [13].

### 2.4. Analysis of the Deterioration Mechanism of the Composite Cross-Arm Silicone Rubber Sheath

After the composite cross-arm is aged, although the high-temperature vulcanized silicone rubber sheath has certain aging resistance, long-term exposure to ultraviolet radiation and salt spray corrosion deteriorates the insulation properties of the material itself. To analyze the deterioration mechanism of the silicone rubber outer sheath, hydrophobicity tests, scanning electron microscopy tests and Fourier transform infrared spectroscopy tests were carried out on the aged outer-sheath samples.

#### 2.4.1. Hydrophobic Angle Measurement

In this paper, the static contact angle method (CA method) was used to measure the hydrophobic angle of the silicone rubber outer sheath before and after aging [14,15]. The aged composite cross-arm outer sheath was selected and then cut into sample pieces with a size of 3 cm × 10 cm and a thickness of 5 mm. Three samples were made for each type of aged composite cross-arm outer sheath, the static contact angles of five water droplets were tested for each sample, and the average value was taken. When the static contact angle θ_av_ ≥ 100° of the sample, it indicates good hydrophobicity; if θ_av_ < 90°, it indicates a temporary loss of hydrophobicity.

#### 2.4.2. SEM Analysis

To observe the degree of surface deterioration of the composite cross-arm silicone rubber outer sheath, the Nova Nano SEM450 scanning electron microscope can be used to conduct microscopic analysis of the silicone rubber outer sheath [16,17]. The samples were rinsed with water and then dried, and the surface state of the outer sheath of the composite cross-arm of different aging types was observed.

#### 2.4.3. FTIR Analysis

Fourier transform infrared spectroscopy (FTIR) is one of the effective means to analyze the chemical reaction of the silicone rubber outer sheath during the aging process [18,19,20]. The main characteristic absorption peaks of the silicone rubber sheath are shown in Table 5. (The absorption peak represents the peak curve in the image of the change of absorption rate with the wave number. If the absorption peak appears at the corresponding wave number, it can be determined that the corresponding characteristic functional group appears).

This paper took samples from the aged T-shaped and square-shaped composite cross arm sheaths for FTIR test. Since the outer sheaths of the two composite cross arms were both made of silicone rubber, the characteristic functional groups that characterize its aging were similar, so Si-O-Si, CH_3_, Si-CH_3,_ and -OH were selected as the main functional groups. By calculating the peak absorption area of the characteristic group, the aging degree of silicone rubber could be analyzed quantitatively. (The area of the absorption peak is obtained by the graphic integration of the absorption peak curve and the abscissa. The larger the area of the absorption peak represents the more characteristic functional groups corresponding to the wave number of the sample, and the smaller the area, the less the content of functional groups.).

## 3. Results and Discussion

### 3.1. Analysis of Artificial Contamination Test Results of Composite Cross-Arms after Aging

#### 3.1.1. Analysis of Leakage Current Test Results

**Figure 4:**
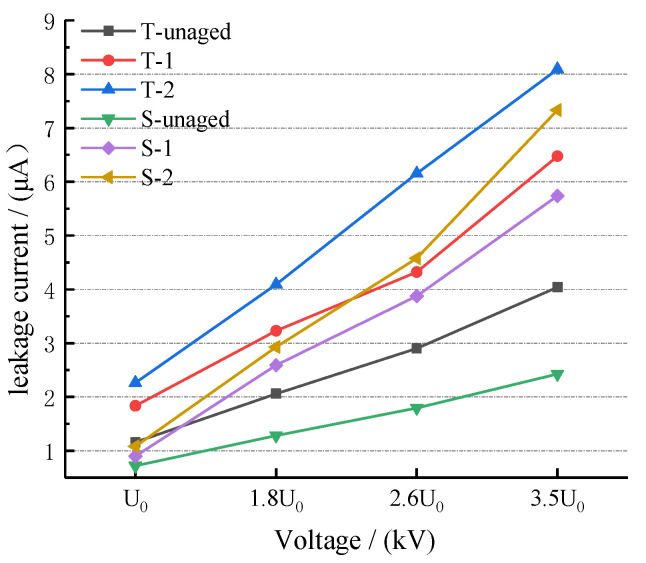
Variation of leakage current of composite cross-arms with different aging factors with applied voltage.

The leakage currents of the square and T-type composite cross-arms at different voltages are shown in Table 6 and Figure 4.

With the increase of the test voltage, the rate of the leakage current of the aged composite cross-bar was greater than that of the unaged composite cross-bar. After comparing the composite cross-arms of the different aging types, it was found that the leakage current value of the cross-arm subjected to ultraviolet aging is generally greater than that of the cross-arm without ultraviolet aging (T-2 > T-1; S-2 > S-1). The reason for this trend is that the hydrophobicity (hydrophobicity is a technical index reflecting the water penetration resistance of materials. The higher the hydrophobicity is, the less water is likely to invade the materials, and vice versa) of the composite cross-arm’s silicone rubber sheath is reduced by ultraviolet radiation; the water absorbed by the surface fouling layer increases to form a continuous water film. The leakage current flows along this conductive path; thus, the conductivity of the composite cross-arm surface increases.

After comparing the two composite cross-arms under the same aging factors, it was found that the leakage current value of the T-shaped cross-arm is generally greater than that of the square-shaped composite cross-arm (T-1 > S-1; T-2 > S-2). The main reason is that the silicone rubber surface area of the T-shaped cross-arm is larger than that of the square-shaped cross-arm, and the surface is more conductive, resulting in a higher leakage current value.

#### 3.1.2. Analysis of Flashover Voltage Test Results

**Figure 5:**
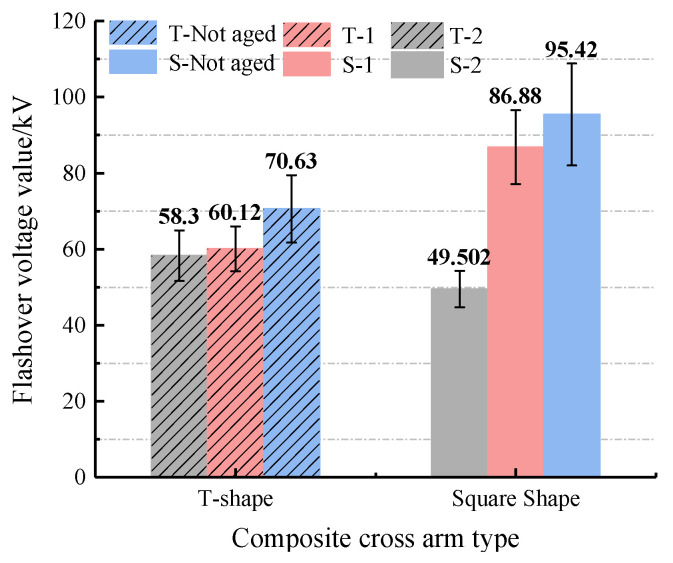
Multi-factor aging composite cross-arm flashover voltage.

Figure 5 shows the change of the flashover voltage before and after the multi-factor aging of the composite cross-arm’s insulation. It can be seen from the trend in the figure that T-2 < T-1 < T-unaged and S-2 < S-1 < S-unaged, indicating that aging factors such as rain, salt spray, damp heat and ultraviolet rays reduce the composite cross-arm’s flashover voltage value. For the T-type cross-arm, T-2 < T-1, so the flashover voltage will further decrease after the composite cross-arm is irradiated with ultraviolet light. For the square cross-arm: S-2 < S-1, which shows a similar law to the T cross-arm. However, for the square cross-arm, the S-2 flashover voltage value decreases more; the S-2 flashover voltage value is only 56% of the S-1 flashover voltage value. Combined with the change law of flashover voltage after aging of the two kinds of composite cross-arms, the reason for analyzing T-2 < T-1 and S-2 < S-1 is because ultraviolet radiation will deteriorate the silicone rubber outer sheath, and the hydrophobicity transference of the outer sheath is weakened. When the soluble substance in the dirt dissolves in water to become electrolytes, and as the wetting reaches saturation, the surface conductance of the outer sheath will be much greater than when it is dry, and this is more likely to cause cross-arm flashover along the surface.

By comparing the flashover voltage values of the two composite cross-arms, it was found that the flashover voltage value of S-1 is higher than that of T-1. Since the composite cross-arm S-1 and T-1 are not subjected to ultraviolet aging, it shows that the outer sheath of the square cross-arm has better resistance to salt spray and damp heat. However, the flashover voltage S-2 of the square composite cross-arm under ultraviolet irradiation is lower than T-2. At the same time, the flashover voltage value of S-2 is much smaller than that of S-1, while the flashover voltage values of T-2 and T-1 are not much different. This shows that the T-type composite cross-arm silicone rubber outer sheath has better UV resistance.

### 3.2. Ultraviolet Discharge Imaging Characteristics of Composite Cross-Arms after Multi-Factor Aging

**Figure 6:**
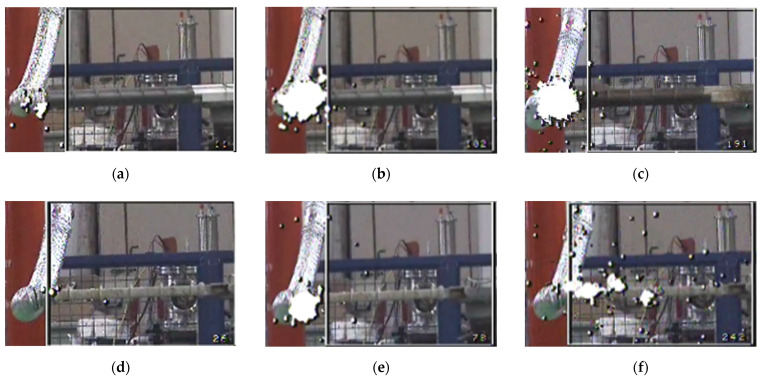
Observation diagram of ultraviolet camera for artificial pollution flashover test of composite cross arm: (**a**) T-unaged; (**b**) T-1; (**c**) T-2; (**d**) S-unaged; (**e**) S-1; (**f**) S-2.

Figure 6 is the UV discharge image of a T-shaped and square composite cross arm at 30 kV voltage.

It can be seen from the figure that the number of discharge spots of the composite cross-arms T-1 and T-2 are significantly higher than that of the unaged cross-arm T-unaged. The higher the number of discharge spots, the stronger the discharge phenomenon on the surface of the cross-arm captured by the UV imager. Compared with the unaged cross-arm, the discharge spots of the aged composite cross-arm are significantly increased under the same voltage, indicating that the surface of the aged composite cross-arm is more prone to discharge, and the external insulation performance is reduced. Comparing T-1 and T-2, it was found that the amount and intensity of discharge spots of the two aging transverse arms are similar. The discharge spots are mainly concentrated at the high voltage end, and no obvious ultraviolet discharge spot was found in other parts of the composite cross arm; this phenomenon is consistent with the change trend of the flashover voltage of the T-type composite cross-arm in Figure 5. In contrast, the number of discharge spots of the square cross-arms S-1 and S-2 are also greater than that of the unaged cross-arm S-unaged, but the range of S-2 discharge spots is wider than S-1, and the discharge is not only concentrated at the high-voltage end. S-2′s discharge development is faster and was able to reach the third section of the umbrella skirt position; therefore, it is more prone to flashover. Comparing the composite cross-arm of the same aging type, it was found that the discharge phenomenon of the composite cross-arm T-1 was stronger than that of S-1, while the discharge phenomenon of T-2 was weaker than that of S-2, indicating that UV irradiation has a significant influence on the external insulation performance of the square composite cross-arm. This phenomenon is consistent with the previous chapter’s flashover voltage test results. If the voltage continues to increase, the discharge phenomenon will continue to develop from the high-voltage end to the fixed hardware in the middle of the cross-arm, eventually leading to flashover of the composite cross-arm.

### 3.3. Microscopic Test Analysis of Composite Cross-Arm Silicone Rubber Sheath

#### 3.3.1. Hydrophobic Angle Test Results and Analysis

**Figure 7:**
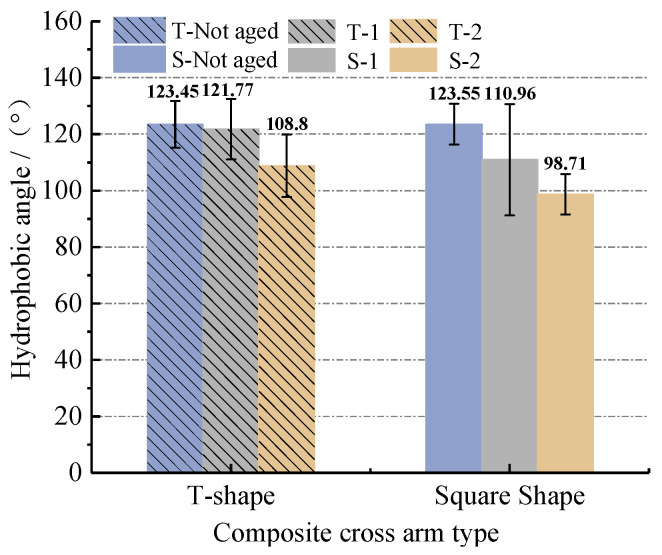
Measurement results of hydrophobic angle of composite cross-arm silicone rubber with different aging types.

Figure 7 shows the test results of the hydrophobic angle of the silicone rubber on the surface of the composite insulating cross-arm with different aging types.

Comparing samples S-1 and S-unaged and T-1 and T-unaged, it was found that the hydrophobic angle of S-1 and T-1 decreased, but both remained above 100° and still maintained good hydrophobicity. This shows that aging factors such as rain, salt spray, damp heat and mechanical force have little effect on the hydrophobicity of the silicone rubber outer sheath. Simultaneously observing samples S-2 and S-unaged and T-2 and T-unaged found that the hydrophobic angles of S-2 and T-2 decreased significantly, and the average value dropped to 98.71° and 108.8°, respectively. This shows that the decrease of the hydrophobic angle of the composite cross-arm will further increase after ultraviolet irradiation. UV light has become the most severe aging factor affecting the hydrophobicity of the outer sheath among many other aging factors. This explains the test results in Section 3.1.1 and Section 3.1.2 of this paper: that is, that S-2 and T-2 have a larger leakage current and a lower pollution flashover voltage than the rest of the aged composite cross-arms.

#### 3.3.2. SEM Analysis

**Figure 8:**
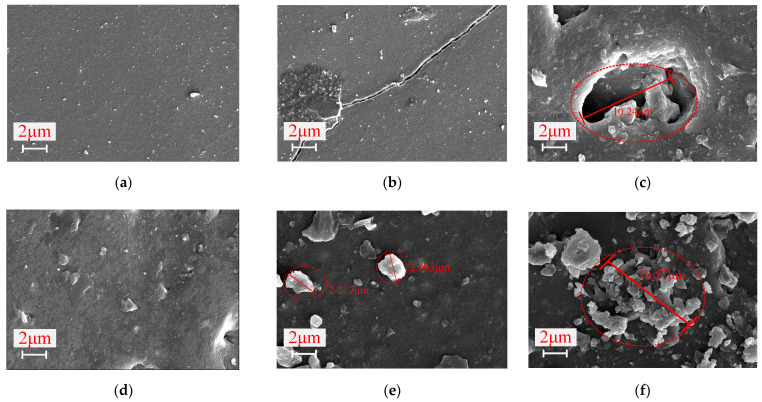
Scanning electron microscope results of the outer surface of the silicone rubber sheath: (**a**) S-unaged; (**b**) S-1; (**c**) S-2; (**d**) T-unaged; (**e**) T-1; (**f**) T-2.

The SEM test results of the composite cross-arm outer sheath with different aging types are shown in Figure 8.

It can be seen from Figure 8 that the surface structure of the unaged cross-arm S-unaged and T-unaged samples is smooth and uniform, without any obvious holes or gaps. The surface of the outer sheath of the composite cross-arm S-1 is smooth, and compared with the unaged cross-arm S-unaged, long cracks are present but no obvious precipitates are found. This shows that aging factors such as rain, salt spray, humidity and high temperatures will cause slight cracking of the square cross-arm silicone rubber but will not affect its internal molecular structure. On the other hand, the diameter of small-diameter particles precipitated on the surface of T-1 is about 2.2 μm. Compared with S-1, the T-1 silicone rubber sheath has undergone molecular changes. In contrast, the surface of S-2 is rough, with ~10.24 μm holes in the center. The pores of the composite cross-arm S-2 silicone rubber show a concave shape from the surrounding surface to the inside, and the aging shows a trend of extending to the inside of the material. Particles with a diameter of about 10.57 μm appeared on the sheath surface of the composite cross-arm T-2, and the density was higher than that of T-1, but no holes or cracks were found.

After the silicone rubber outer sheath is aged by different factors, it shows differences in the microscopic morphology. Among them, the surface of the square cross arm S-2 outer sheath is the most severely aged; there are sunken holes and derivative lines, indicating that the material characteristics of the square cross arm outer sheath have changed after being affected by UV aging. UV irradiation accelerates the deterioration of the sheath material, resulting in a decrease in the overall performance of the material.

#### 3.3.3. FTIR Spectral Analysis

**Figure 9:**
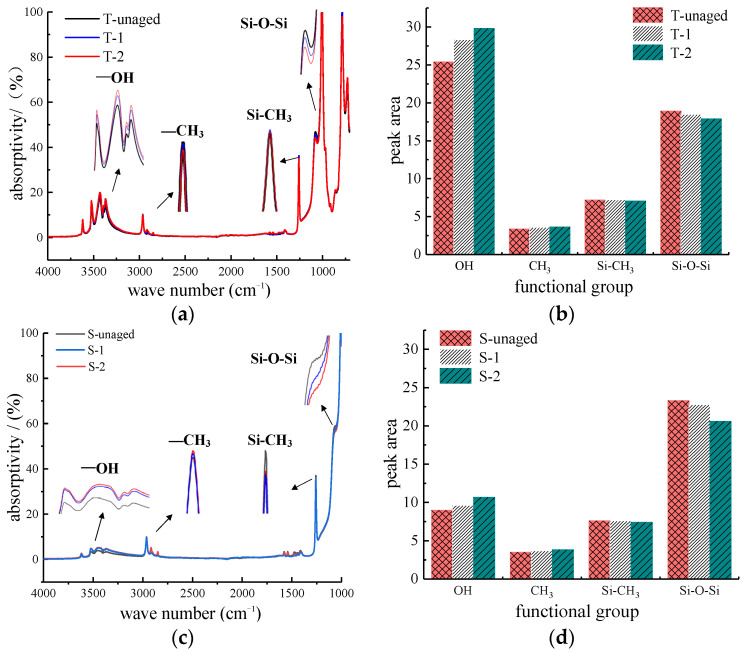
FTIR spectra of T-shaped and square-shaped composite cross-arm silicone rubber sheaths and absorption peak areas of main functional groups: (**a**) T-type FTIR spectrum; (**b**) T-type functional group absorption peak area; (**c**) Square FTIR spectrum; (**d**) Square functional group absorption peak area.

An analysis was conducted of the infrared spectrum of the T-shaped (Figure 9a) and square-shaped (Figure 9c) composite cross-arms. Si-O-Si represents the main chain of silicone rubber; Si-CH_3_ represents the side chain methyl group. If the number of main chain and side chain groups are large and complete, then the silicone rubber sheath has good aging resistance. When observing the absorption peak areas of the main chain group Si-O-Si and the hydrophobic functional group Si-CH_3_ (Figure 9b,d), it can be seen that the main chain group Si-O-Si and hydrophobic group Si-CH3 of the composite arms T-2 and S-2 have the smallest absorption peak areas, and T-unaged and S-unaged have the largest absorption peak areas (T-2 < T-1 < T-unaged; S-2 < S-1 < S-unaged). This shows that after multiple factors age the silicone rubber sheath, the molecular structure inside the material is destroyed. However, the absorption peak area of the hydrophilic group (-OH) with a wavenumber of 3200–3700 cm^−1^ increased after the composite cross-arm was aged, and the -OH absorption peak area of T-2 and S-2 is the largest (T-2 > T-1 > T-unaged; S-2 > S-1 > S-unaged). The increase in the content of hydrophilic groups (-OH) represents the decrease in the hydrophobicity of the outer sheath. This phenomenon occurs because multi-factor aging will break the chemical bond Si-C, under the action of oxygen in the air, the hydrophilic group (-OH) is re-formed. Moreover, Al (OH)_3_ will also decompose to form Al_2_O_3_ and crystal water, increasing in the content of (-OH) groups. At the same time, the absorption peak of CH_3_ located at the wavenumber of 2960 cm^−1^ has a similar trend to the absorption peak of (-OH). This is because the main bond of the silicone rubber has high energy and is not easy to break. The low side chain bond energy causes some methyl groups to easily fall off after multi-factor aging so that the absorption peak of CH_3_ increases. Furthermore, the methyl group, after falling off, undergoes a cross-linking reaction with the main chain, resulting in an increased polarity of the silicone rubber surface; it is more likely to cause flashover of the cross-arm. In addition, UV light caused the most severe deterioration of the outer sheath of the composite cross-arm S-2 and T-2. Ultraviolet light, a high-energy radiation, ionizes the silicone rubber molecules on the surface of the sheath after irradiation, resulting in a higher degree of damage to the silicone rubber. To sum up, after the composite cross-arm is aged by rain, salt spray, ultraviolet, and other factors, the main chain Si-O-Si in the sheath material is broken, the hydrophobic group Si-CH_3_ decreases, the hydrophilic group -OH increases and the insulating performance of the silicone rubber sheath decreases.

## 4. Conclusions

This paper selected two 10 kV composite insulating cross-arms with different cross-sectional shapes. After 5000 h of multi-factor aging testing, the external insulation properties of the composite cross-arm and the deterioration mechanism of the silicone rubber sheath were studied, and the following conclusions were obtained:

1. Compared with before aging, the T-type and square-type composite cross-arms after multi-factor aging have a higher leakage current rising rate and a lower flashover voltage. The external insulation characteristics of the two distribution network composite cross-arms decreased but still retained a certain margin to meet the operating requirements;
2. The SEM test showed that there were pits and cracks on the surface of the sheath, accompanied by precipitates; the hydrophobicity test showed that the hydrophobic angle of the outer sheath of the composite cross-arm decreased to 84% of the initial hydrophobic angle on average; the FTIR test found that the silicone rubber molecules were cracked and increased hydrophilic groups; the microscopic test results showed that the insulation properties of the silicone rubber sheath deteriorate after multi-factor aging. This is the main reason for the decline in the external insulation performance of the composite cross-arm.

In this paper, the influence of various aging factors such as damp heat, rain, salt spray, ultraviolet and mechanical stress on the external insulation performance of the distribution-network composite cross-arm was measured through experiments. Moreover, the reasons for the decline of the insulation performance of the composite cross-arm sheath were analyzed. This provides a theoretical basis for evaluating the operating life of the composite cross-arm in the distribution network.

## Figures and Tables

**Table 1:** 10 kV Composite Cross-Arm Structural Parameters.

Parameter	Square Composite Cross Arm	T-Shaped Composite Cross Arm
Structure length/mm	1750	1860
Section size/mm	h1 = 32, h2 = 42	h1 = 35, h2 = 73
Umbrella skirt height/mm	h3 = 4	h3 = 4
Number of umbrella skirts	5	7
Umbrella skirt spacing/mm	115	95
Creepage distance/mm	1200	1386
Composite cross arm surface area/cm^2^	2660	4181

**Table 2:** Composite cross arm 5000-h multi-factor aging test cycle program.

Period	0–2	2–4	4–6	6–8	8–10	10–12	12–14	14–16	16–18	18–20	20–22	22–24
Humidification			√						√			
High temperature		√	√				√	√				√
Rain wet	√											
Salt spray				√	√				√	√		
UV	√	√	√				√	√	√			
Mechanics				√					√	√		
Voltage	√	√	√	√	√	√	√	√	√	√	√	√

**Table 3:** Multi-factor Aging Subsystem Design Parameters.

Aging Factor	Parametric Design
Humidity	98% RH
Temperature	50 °C ± 0.5 °C
Rain wet	24 h rainfall 50~100 mm
Salt spray	Particle size: 5~10 μm; NaCl content: 7 kg/m^3^; flow rate: 0.5 kg/m^3^ h
UV	Xenon lamp power: 6 kW; UV wavelength: 290~800 nm; Irradiance: 550 W/m^2^
Mechanics	Horizontal load: 177.89 N; Vertical load: 319.69 N; Mechanical force frequency: 0.6 Hz
Voltage	10 kV

**Table 4:** Composite cross-arm aging sample.

Cross-arm Number				Aging Factor			
T	S	Mechanics	UV	Salt Spray	Rain Wet	Hygrothermal	Voltage
T-unaged	S-unaged	×	×	×	×	×	×
T-1	S-1	√	×	√	√	√	√
T-2	S-2	√	√	√	√	√	√

**Table 5:** The infrared absorption peaks of the main characteristic functional groups of the silicone rubber sheath.

Characteristic Functional Group	Wave Number/cm^−1^
O-H	3700–3200
(C-H) in CH_3_	2960
(C-H) in Si-CH_3_	1270–1255
(C-H) in Si-O-CH_3_	1100–1000
Si-(CH_3_)_2_	840–790

**Table 6:** Leakage current of the composite cross-arms with different aging types.

Cross-Arm Number	Test Voltage/kV			
	U_0_	1.8 U_0_	2.6 U_0_	3.5 U_0_
T-unaged	1.156	2.058	2.9	4.04
T-1	1.834	3.231	4.323	6.474
T-2	2.260	4.091	6.152	8.092
S-unaged	0.781	1.282	1.793	2.424
S-1	0.897	2.591	3.877	5.736
S-2	1.081	2.926	4.576	7.332

## Data Availability

The data presented in this study are available on request from the first authors and corresponding author.
